# Examining Equity Sensitivity: An Investigation Using the Big Five and HEXACO Models of Personality

**DOI:** 10.3389/fpsyg.2015.02000

**Published:** 2016-01-08

**Authors:** Hayden J. R. Woodley, Joshua S. Bourdage, Babatunde Ogunfowora, Brenda Nguyen

**Affiliations:** ^1^Department of Psychology, University of Western OntarioLondon, ON, Canada; ^2^Department of Psychology, University of CalgaryCalgary, AB, Canada; ^3^Haskayne School of Business, University of CalgaryCalgary, AB, Canada

**Keywords:** equity sensitivity, Big Five, HEXACO, benevolence, entitlement, honesty/humility, conscientiousness, agreeableness

## Abstract

The construct of equity sensitivity describes an individual's preference about his/her desired input to outcome ratio. Individuals high on equity sensitivity tend to be more input oriented, and are often called “Benevolents.” Individuals low on equity sensitivity are more outcome oriented, and are described as “Entitleds.” Given that equity sensitivity has often been described as a trait, the purpose of the present study was to examine major personality correlates of equity sensitivity, so as to inform both the nature of equity sensitivity, and the potential processes through which certain broad personality traits may relate to outcomes. We examined the personality correlates of equity sensitivity across three studies (total *N* = 1170), two personality models (i.e., the Big Five and HEXACO), the two most common measures of equity sensitivity (i.e., the Equity Preference Questionnaire and Equity Sensitivity Inventory), and using both self and peer reports of personality (in Study 3). Although results varied somewhat across samples, the personality variables of Conscientiousness and Honesty-Humility, followed by Agreeableness, were the most robust predictors of equity sensitivity. Individuals higher on these traits were more likely to be Benevolents, whereas those lower on these traits were more likely to be Entitleds. Although some associations between Extraversion, Openness, and Neuroticism and equity sensitivity were observed, these were generally not robust. Overall, it appears that there are several prominent personality variables underlying equity sensitivity, and that the addition of the HEXACO model's dimension of Honesty-Humility substantially contributes to our understanding of equity sensitivity.

## Introduction

Within the workplace, the notion of equity plays a key role in our understanding of how people perceive and react to injustice. Within this context, equity theory (Adams, 1963, 1965) posits that individuals aspire to have their ratio of inputs and outcomes to be similar to that of relevant comparison others. In other words, people want what they get (e.g., pay, rewards) to accurately reflect what they put in (e.g., effort, performance), and are both intolerant of under-reward (the outcome is smaller than the input) or over-reward (the outcome is greater than the input). Adams (1965) refers to this desired balance as the “norm of equity.” However, an investigation by Huseman et al. (1985) questioned this “norm,” finding that individuals differed regarding what they perceive to be equitable—some individuals were more input oriented while others were more outcome oriented. In essence, not all individuals prefer an equal ratio of inputs to outcomes. As such, individuals' judgments of fairness and equity are more complicated than initially proposed. Huseman et al. (1987) referred to this individual difference construct as “equity sensitivity.” Formally, equity sensitivity describes individuals' preference regarding their inputs and outcomes ratio and was argued to be key to understanding workplace behaviors, particularly in terms of how people form and react to perceptions of inequity and unfairness in the workplace (Huseman et al., 1987).

Despite the fact that equity sensitivity is often described as being a trait, and plays a role in explaining work outcomes—such as citizenship behaviors (Blakely et al., 2005; Akan et al., 2009) and reward preferences (Miles et al., 1994)—we know little about how this construct fits within our existing understanding of personality. Examining the personality traits related to equity sensitivity can inform our understanding of both broad personality traits as well as equity sensitivity. First, we can gain further insight into the extent to which equity sensitivity is indeed a trait, as well as the extent to which it is encompassed within the broad personality models through which we understand personality. Indeed, recent research has noted the importance of establishing the personality variables associated with equity sensitivity and more broadly to better understand its nomological network (Miller, 2009). Second, research on equity sensitivity can better inform us about when and why individuals with certain personality traits will exhibit certain behaviors. For instance, if individuals high on Conscientiousness are more tolerant of inequity and are more input-oriented, this can help us understand the relations between Conscientiousness and input-oriented behaviors such as organizational citizenship behaviors (Organ and Ryan, 1995).

Given this, the goal of the present set of studies was to investigate the personality traits that underlie equity sensitivity. We present the results of three studies to this effect. Across the three studies, we examine this question using the two most prominent models of personality (i.e., the Big Five and the HEXACO model), two popular measures of equity sensitivity (i.e., the Equity Preference Questionnaire and the Equity Sensitivity Inventory), and both self and peer reports of personality.

### Equity sensitivity

Equity sensitivity is typically measured along a continuum. However, to better understand equity sensitivity, each end of the scale is described in greater depth (Huseman et al., 1987). Those who are high in equity sensitivity place more importance on inputs—what they can give in a situation—and so higher scorers have been labeled “Benevolents.” In contrast, those who score toward the low end of the pole on equity sensitivity place greater importance on outcomes—what they can get in a situation—and are labeled as “Entitleds.” Toward the mid-point are those individuals who adhere more closely to the originally proposed norm of equity—that is, those who desire their inputs and outcomes to be balanced. Huseman and colleagues call these individuals “Equity Sensitives.” In sum, along the continuum, individuals who score high on equity sensitivity lean more toward benevolence, whereas individuals who score low on equity sensitivity lean more toward entitlement.

Research has shown that equity sensitivity relates to a variety of workplace attitudes and behaviors. For instance, Shore et al. (2006) found that higher equity sensitivity scores (i.e., more Benevolent) were related to both higher job satisfaction and organizational commitment, while Miles et al. (1994) found that lower equity sensitivity scores (i.e., more Entitled) were related to individuals' preference for extrinsic outcomes. Further, Mudrack et al. (1999) found that equity sensitivity helped to explain attitudes toward ethics-related criteria (e.g., corporate social responsibility). Lastly, research has shown that equity sensitivity is positively related to both organizational (Blakely et al., 2005) and team (Akan et al., 2009) citizenship behaviors.

Beyond these main effects, much of the research on equity sensitivity has focused on its role as a key moderator of important work relations (e.g., O'Neill and Mone, 1998). For instance, researchers have demonstrated that Entitleds and Benevolents respond differently to breaches in psychological contract depending on the type of outcome associated with the breach. On the one hand, when the breach affected extrinsic outcomes (e.g., pay), Entitleds were more likely than Benevolents to react negatively (Kickul and Lester, 2001). On the other hand, when the breach affected intrinsic outcomes (e.g., autonomy) Benevolents were more likely than Entitleds to react negatively (Restubog et al., 2007). These differential patterns of relations highlight the importance of understanding individual differences in equity sensitivity.

### Equity sensitivity and personality

To date, there is a paucity of research investigating the nomological network of equity sensitivity, especially in regards to broad personality frameworks (e.g., the Big Five and HEXACO models). Often, when researchers have examined the relation between equity sensitivity and personality traits, they tend to focus on specific traits (e.g., Conscientiousness and/or Extraversion; cf. Raja et al., 2004; Scott and Colquitt, 2007). In addition, researchers have tended to report simple bivariate relations between equity sensitivity and these personality traits. As such, we do not know how broad personality traits—when examined together—uniquely predict equity sensitivity. Utilizing a multivariate approach is key to fully understanding and developing the nomological network of equity sensitivity.

To address this research gap, the current research investigates the relations between equity sensitivity and broad personality traits (Big Five and HEXACO) in three studies. In the first study, we examine the relation between equity sensitivity and the “Big Five” personality traits—i.e., Conscientiousness, Agreeableness, Neuroticism, Openness to Experience, and Extraversion (Digman, 1990; McCrae and Costa, 1997). In the second study, we examine whether the measurement of a sixth personality trait—Honesty-Humility from the HEXACO personality model (Lee and Ashton, 2004)—adds to the prediction of equity sensitivity beyond the Big Five traits In the third study, we expand on this by utilizing the entirety of the HEXACO model, and determining the extent to which our findings generalized using peer-reports of personality and two distinct measures of equity sensitivity.

## Study 1

### The big five

In the first study, we began by investigating how equity sensitivity relates to the “Big Five” personality traits. The Big Five were selected because they are arguably the most widely researched personality traits. Although previous research has examined the relation between some of the Big Five traits (e.g., Conscientiousness and Agreeableness; Konovsky and Organ, 1996; Bing and Burroughs, 2001; Shore and Strauss, 2008) and equity sensitivity, we are unaware of any investigations that have examined the relations between all five traits and equity sensitivity simultaneously. Thus, to contextualize equity sensitivity within our existing understanding of personality, we first sought to investigate its relation with the Big Five personality traits.

#### Conscientiousness

Individuals who score high in Conscientiousness are considered to be methodical, organized, productive, and tend not to self-indulge (Digman, 1990; McCrae and John, 1992). In the workplace, conscientious individuals tend to work hard (i.e., input to their workplace) even when extrinsic outcomes (e.g., pay) are low (Burnett et al., 2009). Similarly, conscientious individuals also engage in organizational citizenship behaviors, which capture the extent to which one goes over and above allocated job requirements without promise of additional compensation or recognition (e.g., *giving* a helping hand to a coworker; attending corporate functions). In other words, conscientious individuals tend to engage in benevolent, discretionary actions in the workplace (Konovsky and Organ, 1996; Bowling, 2010). Not surprisingly, a number of research studies (Konovsky and Organ, 1996; Bing and Burroughs, 2001; Raja et al., 2004; Scott and Colquitt, 2007) have found Conscientiousness to be positively related to equity sensitivity (i.e., more Benevolent). We therefore, hypothesized:

*Hypothesis 1: Conscientiousness will be positively related to equity sensitivity (i.e., more Benevolent)*.

#### Agreeableness

Individuals who score high in Agreeableness are considered to be altruistic, caring, selfless, and helpful (McCrae and Costa, 1987; Digman, 1990). Agreeable individuals tend to help both supervisors and coworkers (Kamdar and Van Dyne, 2007) and generally engage in organizational citizenship behaviors at work (Ilies et al., 2009; Chiaburu et al., 2011). Further, agreeable individuals tend to have lower income despite their profession (Ng et al., 2005; Judge et al., 2012). This lower income may be the result of poor bargaining tactics. Barry and Friedman (1998) found that Agreeableness is negatively related to distributive bargaining (e.g., for compensation) because agreeable individuals place greater important on interpersonal relations and less on outcomes. In sum, previous research findings suggest that agreeable individuals may be more input oriented and less outcome-oriented; thus, they would score high on equity sensitivity. Commensurate with these findings, Bing and Burroughs (2001), Konovsky and Organ (1996), and Scott and Colquitt (2007) all found Agreeableness to be positively related to equity sensitivity (i.e., more Benevolent). Therefore, we hypothesized:

*Hypothesis 2: Agreeableness will be positively related to equity sensitivity (i.e., more Benevolent)*.

#### Neuroticism

The relation between Neuroticism and equity sensitivity is less clear. Individuals who score high in Neuroticism (i.e., Emotional Stability) tend to be shy, worrisome and anxious (McCrae and John, 1992). Research examining Neuroticism in the workplace has found that neurotics perceive themselves as being targets of incivility (Milam et al., 2009), have lower job satisfaction (Judge et al., 2002), are more likely to report feeling their psychological contract has been breached (Raja et al., 2004), and perform more counterproductive work behaviors (Berry et al., 2007). It is possible that, as a result of these perceptions, neurotic individuals will be more likely to withdraw (i.e., reduce their inputs) in the workplace. Hence, there is reason to suspect a small relation between Neuroticism and equity sensitivity. This is supported by two studies that have shown fairly modest associations between Neuroticism and equity sensitivity—individuals high in Neuroticism are more likely to score lowly on equity sensitivity (i.e., more Entitled; Raja et al., 2004; Scott and Colquitt, 2007). Thus, the following is hypothesized:

*Hypothesis 3: Neuroticism will be negatively related to equity sensitivity (i.e., more Entitled)*.

#### Openness to experience

Individuals who score high in Openness to Experience are described as imaginative, liberal in their values, flexible in regards to norms on how they behave, and appreciate both intellectual stimulation and art (McCrae and Sutin, 2009). Interestingly, a growing body of evidence indicates that individuals high on Openness may have a tendency toward increased input relative to outcomes (i.e., high equity sensitivity). For instance, Openness has shown to be related to empathy (McCrae and Costa, 1997), altruism (Landis et al., 2009), and prosocial values (Carlo et al., 2005). In terms of broader social values (Schwartz, 1992), individuals high in Openness also tend to endorse Self-Transcendent values, such as benevolence and universalism (Lee et al., 2010). Such values may translate into input-oriented workplace behavior. For instance, Bourdage et al. (2012) found Openness to be positively related to organizational citizenship behaviors, in particular those motivated by prosocial values. In addition, Liao et al. (2008) found Openness to be negatively related to work withdrawal, which implies that individuals who are high in Openness are less likely to withdraw (i.e., withhold input) in the workplace. As a result of these findings, the following is hypothesized:

*Hypothesis 4: Openness to Experience will be positively related to equity sensitivity (i.e., more Benevolent)*.

#### Extraversion

Individuals who score high in Extraversion are considered to be sociable, outgoing, and fun loving (McCrae and Costa, 1987). In regards to equity sensitivity, research by Chiaburu et al. (2011) found a small, positive relation between Extraversion and organizational citizenship behavior. Further, both Bolton et al. (2010) and Liao et al. (2008) found extraverts to be less likely to exhibit withdrawal behaviors in the workplace. These findings indicate that extraverts are likely to be input oriented. Yet, extraverts have also been described as being reward driven (Gray, 1981). For example, Extraversion has been positively related to outcomes such as career success and income (Judge et al., 1999; Seibert and Kraimer, 2001; Ng et al., 2005), indicating that extraverts may also be outcome oriented. Even further, research directly investigating Extraversion and equity sensitivity has found a positive relation (i.e., more Benevolent; Raja et al., 2004; Colquitt et al., 2006; Scott and Colquitt, 2007). Nevertheless, other measures of entitlement (e.g., psychological entitlement and narcissistic entitlement) have found either no relation or a *positive* relation with Extraversion (e.g., Campbell et al., 2004; Donnellan et al., 2006; Pryor et al., 2008), suggesting that Extraversion would be either unrelated or possibly negatively related to equity sensitivity (i.e., more Entitled). In sum, there is not enough convincing evidence to hypothesize a directional relation between these two constructs. As such, we investigate the relation between equity sensitivity and Extraversion in an exploratory fashion.

Research Question 1: What is the nature of the relation between Extraversion and equity sensitivity?

#### Full model

As we note above, previous research investigating the relation between Big Five traits and equity sensitivity (e.g., Raja et al., 2004; Scott and Colquitt, 2007) have not included all of the Big Five traits, nor have they conducted any multivariate analyses to examine how each trait uniquely predicts equity sensitivity. One goal of the present study is to address this research gap by examining the unique contribution of each of the personality traits, and to determine the combined overlap of broad personality with equity sensitivity. We argue that certain personality traits may relate to equity sensitivity for differing reasons. For example, and as previously discussed, conscientious individuals are prone to perform input-oriented behaviors, whereas agreeable individuals may be less concerned with outcomes. Both of these traits would therefore be positively related to equity sensitivity, but their contributions to predicting equity sensitivity may be unique. Nonetheless, there is not enough evidence to hypothesize which of the personality traits may or may not uniquely predict equity sensitivity when taking all of the Big Five traits into account in a multivariate analysis. Thus, we sought to answer the following research question:

Research Question 2: Which of the Big Five traits predicts unique variance in equity sensitivity?

### Methods

#### Participants and procedure

Participants were undergraduate students enrolled in a first year psychology course at a large Canadian university. The sample consisted of 499 participants, with a mean age of 18 years (range: 16–42) and predominately female (60%) with a majority ethnicity of Caucasian (63%). In accordance with the university's Non-Medical Research Ethics Board, all participants provided electronic informed consent prior to participating in Study 1. Through an online testing process, participants completed a battery of questionnaires to earn course credit. Participants were provided instructions for each questionnaire they completed.

#### Measures

##### Equity sensitivity

Equity sensitivity was measured using the Equity Preference Questionnaire (EPQ) developed by Sauley and Bedeian (2000). High scores on the EPQ describe a tendency toward Benevolence, whereas low scores describe a tendency toward Entitlement. The EPQ consists of eight positively keyed Benevolent items and eight negatively keyed Entitled items. An example of a Benevolent item is, “I feel obligated to do more than I am paid to do at work.” An example of an Entitled item is, “When I am at my job, I think of ways to get out of work.” These items were responded to on a five-point Likert-type scale (1 = strongly disagree to 5 = strongly agree). Researchers that have utilized the EPQ have generally found the measure to demonstrate strong internal consistency (e.g., Shore and Strauss, 2008). In regards to scoring, some research has questioned the dimensionality of the EPQ (e.g., Miller, 2009), with Benevolent and Entitled items loading on different factors. However, this effect could be the result of having positively and negative worded items (see Spector et al., 1997). In addition, the vast majority of the research using the EPQ has scored the construct unidimensionally. Thus, based on the findings of Spector et al. (1997), and to be consistent with previous research, we treated the EPQ as a unidimensional scale^1^.

##### Big five

The Big Five personality traits were measured using items from the International Personality Item Pool (IPIP; Goldberg et al., 2006). A 50-item measure–10 items per trait—based on Costa and McCrae's (1992) NEO PI-R, was used from the IPIP. For each trait, there were five positively keyed and five negatively keyed items. These items were rated on a five-point Likert-type scale (1 = strongly disagree to 5 = strongly agree). Goldberg et al. (2006) report strong internal reliability for these scales with Cronbach's alphas ranging from 0.77 to 0.86.

### Results

The means, standard deviations, intercorrelations and Cronbach's alphas for all variables are reported in Table 1. Hypothesis 1, which stated that Conscientiousness would be positively related to equity sensitivity, was supported (*r* = 0.42, *p* < 0.01). Hypothesis 2, which stated that Agreeableness would be positively related to equity sensitivity, was supported (*r* = 0.40, *p* < 0.01). Hypothesis 3, which stated that Neuroticism would be negatively related to equity sensitivity, was not supported (*r* = −0.05, ns). Hypothesis 4, which stated that Openness would be positively related to equity sensitivity was supported (*r* = 0.15, *p* < 0.01). Lastly, in regards to Research Question 1, Extraversion was unrelated to equity sensitivity (*r* = 0.03, ns).

**Table 1:** **Variable means, standard deviations, intercorrelations, and Cronbach's alphas for Study 1**.

**Variable**	***M***	***SD***	**1**	**2**	**3**	**4**	**5**	**6**
1. EPQ	3.53	0.63	(0.89)^a^					
2. C	3.36	0.64	0.42^**^	(0.78)^a^				
3. A	3.62	0.54	0.40^**^	0.28^**^	(0.76)^a^			
4. N	2.61	0.70	−0.05	−0.33^**^	−0.33^**^	(0.82)^a^		
5. O	3.47	0.60	0.15^**^	0.12^*^	0.24^**^	−0.05	(0.75)^a^	
6. E	3.54	0.73	0.03	0.19^**^	0.08	−0.31^**^	0.21^**^	(0.88)^a^

*M, mean; SD, standard deviation; EPQ, Equity Preference Questionnaire; C, Conscientiousness; A, Agreeableness; N, Neuroticism; O, Openness to Experience; E, Extraversion*.

** p < 0.01;

* *p < 0.05*.

a *Cronbach's alpha*.

#### Multiple regression analysis

In order to test our second research question, we ran a multiple regression analysis to determine the unique contribution of each trait in predicting equity sensitivity, as well as the extent to which the Big Five traits combined to account for equity sensitivity (see Table 2). Overall, the Big Five model accounted for a significant proportion of variance in equity sensitivity (*R*^2^ = 0.29, *p* < 0.01). The results further show that Conscientiousness (β = 0.38, *p* < 0.01) and Agreeableness (β = 0.34, *p* < 0.01) predicted unique variance in equity sensitivity. Interestingly, Neuroticism (β = 0.19, *p* < 0.01) also predicted unique variance in equity sensitivity, with high scorers in Neuroticism being more likely to be Benevolent. This was contrary to the proposed theory; however, due to the near zero correlation between these two variables, this may be an artifactual relation resulting from the presence of much stronger predictors in the equation or the result of suppression effects.

**Table 2:** **Summary of multiple regression and relative weights analysis for Study 1**.

**Variables**	**β**	***SE***	***t***	**rRW**	**RW%**	**CI_*L*_**	**CI_U_**
C	0.38^**^	0.05	8.43^**^	0.15	51.54	0.08	0.22
A	0.34^**^	0.05	7.27^**^	0.12	41.12	0.06	0.18
N	0.19^**^	0.04	4.04^**^	0.01	3.98	−0.00^a^	0.03^a^
O	0.04	0.05	0.82	0.01	2.95	−0.01^a^	0.03^a^
E	0.02	0.04	0.39	0.00	0.42	−0.02^a^	0.01^a^
R^2^	0.29^**^						

*β, standardized regression weight; SE, standard error; R^2^, squared multiple correlation; rRW, raw relative weight; RW%, relative weight percentage; CI_L_ and CI_U_, lower and upper bounds, respectively, of the 95% confidence interval for the significance test. C, Conscientiousness; A, Agreeableness; N, Neuroticism; O, Openness to Experience; E, Extraversion*.

** *p < 0.01*.

a *Confidence intervals that contain zero are considered to be non-significant*.

*Given that this finding is contrary to the limited evidence to date (e.g., Scott and Colquitt, 2007), we caution against substantive interpretation of this finding*.

##### Relative importance analysis

We conducted a relative importance analysis to examine which of the Big Five personality traits accounted for the most variance in equity sensitivity. This technique allows for a clearer understanding of the relation between the multiple predictors and the outcome variable, especially when predictors are correlated. Relative importance analysis partitions the variance accounted for (i.e., the *R*^2^) by the regression model between the included predictors to examine how much each predictor contributed to the variance in the criterion variable (for more information regarding this analysis and its advantages over other techniques, see Johnson, 2000; Johnson and LeBreton, 2004; Tonidandel and LeBreton, 2011).

We conducted the relative importance analysis using (Tonidandel and LeBreton, 2014) web-based tool. The results of this analysis are presented in Table 2. The analysis revealed that, of the five traits, only Conscientiousness and Agreeableness accounted for a significant portion of the total variance in equity sensitivity, with ~92% of the variance being associated with the two personality traits.

### Discussion

The results of Study 1 found general support for our hypotheses regarding the relation between the Big Five personality traits and equity sensitivity. In agreement with the findings of previous research (e.g., Konovsky and Organ, 1996; Bing and Burroughs, 2001; Scott and Colquitt, 2007), we found support for positive relations between both Conscientiousness and Agreeableness with equity sensitivity. These findings were further corroborated through multiple regression and relative importance analyses, demonstrating that Conscientiousness and Agreeableness both predicted unique variance in equity sensitivity as well as accounted for the majority of total variance accounted for by the Big Five model.

Our hypothesis regarding Neuroticism was not supported. We observed a non-significant zero-order relation between Neuroticism and equity sensitivity. In addition, a significant and *positive* relation was found in our regression analyses. The relative weights analysis, however, showed that Neuroticism did not explain a significant amount of variance in equity sensitivity compared to the other Big Five traits. We therefore, concluded this positive relation to be spurious. However, we still sought to further investigate the nature and generalizability of this relation in Studies 2 and 3.

We also found that Openness to Experience was positively related to equity sensitivity. However, it did not predict unique variance in equity sensitivity nor a significant portion of the variance accounted for in the regression model. Thus, only partial support was found for Hypothesis 4. Although, there does seem to be a positive relation between Openness and equity sensitivity, this relation is not significant when accounting for other personality variables. Last, Extraversion was not found to have any relation to equity sensitivity—neither correlational nor in the regression model. Further, Extraversion accounted for less than 1% of the variance in equity sensitivity in the regression model.

In sum, the results of our first study indicate that certain Big Five personality traits are significantly associated with equity sensitivity. We found that conscientious and agreeable individuals tend to score higher on equity sensitivity (i.e., more Benevolent). Interestingly, the Big Five traits—as a whole—did not explain a substantial amount of variance in equity sensitivity (less than 30%). This suggests that equity sensitivity may not be well-encapsulated by the Big Five framework. As such, Study 2 investigated the incremental predictive ability of including a broad personality variable that lies beyond the Big Five—namely, Honesty-Humility of the HEXACO model (Lee and Ashton, 2008).

## Study 2

Despite the prevalence of the Big Five personality traits in the literature, other personality models have been presented over the years that have been able to predict variance unaccounted for by the Big Five (cf. Lee et al., 2005a). Most notably, the HEXACO model of personality (Lee and Ashton, 2004) has emerged as one alternative model of personality that has gained prominence. The HEXACO is named for the six personality traits it represents: Honesty-Humility, Emotionality, Extraversion, Agreeableness, Conscientiousness, and Openness to Experience (Lee and Ashton, 2004). Although, there is quite a bit of overlap between the Big Five and HEXACO models, the most notable addition in the HEXACO model is the personality factor of Honesty-Humility. We predict that Honesty-Humility will add to our understanding of equity sensitivity, as we detail below. In addition, the present study will allow a test of the replicability of the relations between the Big Five and equity sensitivity that emerged in Study 1.

### Honesty-humility

Honesty-Humility captures individual differences in sincerity, modesty, greed avoidance, and fairness (Lee and Ashton, 2008). Research indicates that Honesty-Humility explains unique variance in several traits that are not well-accommodated under the Big Five umbrella, such as self-monitoring personality (Ogunfowora et al., 2013), the Dark Triad personality traits (Paulhus and Williams, 2002; Lee et al., 2013), and several of the traits encompassed in the Supernumerary Personality Inventory (Paunonen, 2002), including seductiveness, manipulativeness, integrity, and egotism (Lee et al., 2005b). Given that ideas about fairness and individual differences in modesty are integral parts of Honesty-Humility, we believe this trait will add to our understanding of equity sensitivity beyond the Big Five.

Individuals who are low on Honesty-Humility tend to believe they are superior to others and that they deserve more in life than others (Lee and Ashton, 2008). As such, they are often driven to attain high status outcomes. These individuals are also willing to manipulate, deceive, and exploit others for their own personal gain and will treat others unfairly if the opportunity arises. Indeed, Hilbig and Zettler (2009) found that individuals who are low in Honesty-Humility are more likely to make selfish decisions regarding reward allocations. This suggests that these individuals, who generally have greater feelings of entitlement, expect to obtain higher outcomes relative to others. This is in line with Lee and Ashton's (2006) theoretical interpretation of this trait. They propose that Honesty-Humility captures a unique form of altruistic tendency; an unwillingness to take advantage of others in interpersonal relationships, even when one stands to gain significantly by doing so. Individuals who are low on Honesty-Humility have little hesitation in this regard. In support of these theoretical arguments, recent research has found that the Dark Triad personality traits are strong predictors of equity sensitivity (Woodley and Allen, 2014). This is particularly relevant given that (low) Honesty-Humility has been found to be the trait underlying the Dark Triad variables (Lee et al., 2013). Thus, individuals low in Honesty-Humility should be more likely to pursue disproportionately larger outcomes relative to others, and believe they deserve these outcomes—an orientation that is consistent with the Entitled end of equity sensitivity. Conversely, individuals high in Honesty-Humility (who believe they are no better than others, are unlikely to take advantage of others, and do not particularly value high status outcomes) would be more likely to score highly on equity sensitivity (i.e., be more Benevolent). Thus, the following is hypothesized:

*Hypothesis 5a: Honesty-Humility will be positively related to equity sensitivity (i.e., more Benevolent)*.
*Hypothesis 5b: Honesty-Humility will add to the prediction of equity sensitivity over and above the Big Five personality traits*.

### Methods

#### Participants and procedure

The sample consisted of 411 undergraduate students enrolled in a first year engineering course at a large Canadian university. The average age of the participants was 19 years (range from 16 to 42), and the majority were male (78%), and Caucasian (62%). On average, participants reported having worked for just over 2 years with a range of 0–17 years of work experience. Only 22% of the participants had no work experience, with 29% of the participants reporting having worked for at least 2 years. In accordance with the university's Non-Medical Research Ethics Board, participants provided written informed consent prior to participating in the following investigation. Data was gathered on the first day of classes. Participants were informed that they would receive a bonus percentage on their final grade for completing a battery of questionnaires. Instructions were provided for each questionnaire within the survey booklet. Questionnaires were administered in a paper-and-pencil format and took ~20 min for participants to complete.

#### Measures

##### Equity sensitivity

Consistent with Study 1, the Equity Preference Questionnaire (EPQ) was used to measure equity sensitivity (Sauley and Bedeian, 2000). Participants responded to the EPQ items on a five-point Likert-type agreement scale (1 = strongly disagree to 5 = strongly agree). High scores on this scale capture Benevolents, whereas low scorers can be considered Entitleds.

##### Big five

The Big Five personality traits were again measured using items from the International Personality Item Pool (IPIP; Goldberg et al., 2006). As with Study 1, these items were designed to reflect the content in Costa and McCrae's (1992) NEO PI-R. However, a much larger battery of items (120 items; 24 per trait) was used with each trait. These 120 items have been utilized in previous research and are both reliable and valid (Hastings and O'Neill, 2009; O'Neill and Allen, 2011).

##### Honesty-humility

The 10 items used to measure Honesty-Humility were taken from Ashton and Lee's (2009) 60-item HEXACO measure. The items were rated on a five-point Likert-type agreement scale (1 = strongly disagree to 5 = strongly agree). Past research has found these items to be quite reliable in both student and community samples (Ashton and Lee, 2009).

### Results

The means, standard deviations, intercorrelations and Cronbach's alphas for all variables are reported in Table 3. Conscientiousness (*r* = 0.45, *p* < 0.01), Agreeableness (*r* = 0.32, *p* < 0.01), Openness to Experience (*r* = 0.14, *p* < 0.01), and Extraversion (*r* = 0.12, *p* < 0.05) were positively related to equity sensitivity, whereas Neuroticism was negatively related (*r* = −0.20, *p* < 0.01). As predicted, Honesty-Humility was positively related to equity sensitivity (*r* = 0.39, *p* < 0.01). These findings are consistent with Hypotheses 1, 2, 3, 4, and 5a.

**Table 3:** **Variable means, standard deviations, intercorrelations, and Cronbach's alphas for Study 2**.

**Variable**	***M***	***SD***	**1**	**2**	**3**	**4**	**5**	**6**	**7**
1. EPQ	3.54	0.58	(0.89)^a^						
2. C	3.69	0.50	0.45^**^	(0.88)^a^					
3. A	3.65	0.44	0.32^**^	0.33^**^	(0.84)^a^				
4. N	2.60	0.53	−0.20^**^	−0.32^**^	−0.17^**^	(0.87)^a^			
5. O	3.15	0.49	0.14^**^	0.04	0.23^**^	−0.09	(0.82)^a^		
6. E	3.46	0.49	0.12^*^	0.12^*^	0.05	−0.44^**^	0.28^**^	(0.87)^a^	
7. H	3.27	0.61	0.39^**^	0.31^**^	0.58^**^	−0.11^*^	0.15^**^	−0.15^**^	(0.72)^a^

*M, mean; SD, standard deviation; EPQ, Equity Preference Questionnaire; C, Conscientiousness; A, Agreeableness; N, Neuroticism; O, Openness to Experience; E, Extraversion; H, Honesty-Humility*.

** p < 0.01;

* *p < 0.05*.

a *Cronbach's alpha*.

#### Multiple regression analysis

A hierarchical multiple regression analysis was conducted to examine whether Honesty-Humility added to the prediction of equity sensitivity beyond the Big Five (see Table 4).

**Table 4:** **Summary of the multiple regression and relative weight analysis for Study 2**.

**Block**	**Variable**	**β_Block 1_**	**β_Block 2_**	**rRW**	**RW%**	**CI_L_**	**CI_U_**
1	C	0.41^**^	0.38^**^	0.16	48.45	0.09	0.24
	A	0.20^**^	0.05	0.05	13.61	0.02	0.09
	N	0.04	0.07	0.01	1.91	−0.01^a^	0.02^a^
	O	0.06	0.03	0.01	2.10	−0.01^a^	0.04^a^
	E	0.07	0.14^*^	0.02	4.65	−0.01^a^	0.05^a^
2	H		0.29^**^	0.10	29.28	0.05	0.16
Overall R^2^		0.28^**^	0.33^**^				
ΔR^2^			0.05^**^				

*β, standardized regression weight; R^2^, squared multiple correlation; rRW, raw relative weight; RW%, relative weight percentage; CI_L_ and CI_U_, lower and upper bounds, respectively, of the 95% confidence interval for the significance test. C, Conscientiousness; A, Agreeableness; N, Neuroticism; O, Openness to Experience; E, Extraversion; H, Honesty-Humility*.

** p < 0.01;

* *p < 0.05*.

a *Confidence intervals that contain zero are considered to be non-significant*.

*sensitivity, ΔR^2^ = 0.05, providing support for hypothesis 5b. Together, the Big Five plus Honesty-Humility captured 33% of the variance in equity sensitivity*.

In step one of the regression, equity sensitivity was regressed on the Big Five traits. In the regression model, only Conscientiousness (β = 0.41, *p* < 0.01) and Agreeableness (β = 0.20, *p* < 0.01) predicted unique variance in equity sensitivity with an overall *R*^2^ = 0.28, *p* < 0.01. These findings are consistent with Study 1. In step two, Honesty-Humility was added to the regression model. In this new model, Conscientiousness (β = 0.38, *p* < 0.01), Extraversion (β = 0.14, *p* < 0.05), and Honesty-Humility (β = 0.29, *p* < 0.01) predicted unique variance in equity sensitivity, with Honesty-Humility incrementally predicting equity sensitivity.

##### Relative importance analysis

Results of the relative importance analysis are presented in Table 4. The analysis revealed that, Conscientiousness, Agreeableness, and Honesty-Humility accounted for a significant portion of the total variance in equity sensitivity. Conscientiousness accounted for the largest portion of variance (48.45%) with Honesty-Humility (29.28%) and Agreeableness (13.61%) accounting for the second and third most variance, respectively.

### Discussion

The results of Study 2 found further support for our hypotheses regarding the relation between the Big Five personality traits and equity sensitivity. As in Study 1, Conscientiousness, Agreeableness, and Openness were positively related to equity sensitivity, providing further support for Hypotheses 1, 2, and 4. Unique to Study 2, Neuroticism was negatively related to equity sensitivity—providing support for Hypothesis 3—and Extraversion was found to positively relate to equity sensitivity, although the findings with Extraversion and Neuroticism were not robust across analyses. Consistent with Study 1, multiple regression and relative importance analysis demonstrated that Conscientiousness and Agreeableness predicted unique variance in equity sensitivity and accounted for the majority of its variance, whereas Neuroticism, Openness, and Extraversion did not. As such, when we take into account the relative and incremental role of personality in predicting equity sensitivity, Conscientiousness and Agreeableness seem to be the most prominent of the Big Five personality traits in understanding equity sensitivity. Beyond the Big Five, however, we found that Honesty-Humility correlated with and accounted for unique variance in equity sensitivity, supporting those respective hypotheses. In addition, the relative importance analysis demonstrated that Honesty-Humility accounted for the second most variance in equity sensitivity. Indeed, this finding indicates that the addition of the Honesty-Humility factor significantly adds to our understanding of the personality bases of equity sensitivity. We found that low Honesty-Humility was related to being more Entitled, whereas high Honesty-Humility was related to being more Benevolent. However, because these findings were again limited to self-reports and to one measure of equity sensitivity, we sought to further examine the robustness of these findings in Study 3.

## Study 3

Study 3 was conducted to address potential limitations of the previous two studies. First, although Study 2 introduced the personality trait of Honesty-Humility as an incremental predictor of equity sensitivity beyond what was found by the Big Five, the full HEXACO personality model was not implemented. In some cases, the HEXACO model as a whole does a better job of predicting criteria than the Big Five plus Honesty-Humility (e.g., Ashton and Lee, 2008; Lee et al., 2013). Indeed, there are important differences between the two models, beyond the presence of the Honesty-Humility factor, including the fact that the Agreeableness and Emotionality factors are rotational variants of their Big Five counterparts. Second, the two previous studies included only self-report data. This can result in common method bias, which can have a negative effect on the validity of the results (Podsakoff et al., 2003). Hofstee (1994) argues that it is important to demonstrate evidence beyond self-report measures when investigating the nature of associations among personality constructs. Moreover, there are likely to be unique aspects of the relations that may go unnoticed if observer reports of personality traits are not incorporated into the validation process (Oh et al., 2011). Finally, in both studies, we used the same measure of equity sensitivity (i.e., the EPQ). While the EPQ is perhaps the most prominent measure of equity sensitivity, to add to the comprehensive nature of this investigation, expanding our investigation of equity sensitivity beyond just the EPQ was necessary.

In sum, the current study investigates the relation between personality and equity sensitivity using the entirety of the HEXACO personality model. In addition, both self-ratings and peer-ratings were obtained for the HEXACO model to examine the robustness of our findings across sources. Finally, we measured equity sensitivity using both the Equity Preference Questionnaire and the Equity Sensitivity Inventory (Huseman et al., 1985). We only utilized self-reports of the equity sensitivity measures, as they refer to workplace content, and the peers in the present study were not required to know the target in a workplace context.

### Methods

#### Participants and procedure

Participants were 260 undergraduate students enrolled in a Canadian undergraduate psychology program. The mean age of participants was 20.64 (*SD* = 3.97), and 63.8% were female, 35.0% were male, and 3% did not report sex. Approximately 58.5% were currently employed, and of those who worked, the average amount worked was 15.52 h per week (*SD* = 10.13). Moreover, 95% of participants had been employed in the past. Students were asked to come into the lab in pairs, and each student completed a self-report and peer-report questionnaire. In accordance with the university's Conjoint Faculties Research Ethics Board, participants provided written informed consent prior to participating in the following investigation. Participants were either seated at separate tables, or separated by a 3-foot tall divider. Participants were ensured that their partners would not see their responses. They were required to have known each other for at least 6 months. Participants were also asked to rate the quality of their relationship with the person they came in with on a scale ranging from 1 to 10. Overall, they rated the quality of their relationship reasonably high (*M* = 7.75, *SD* = 1.59).

#### Measures

##### HEXACO

The HEXACO personality traits were measured using the 100-item versions of the self and observer reports of the HEXACO-PI-R (Lee and Ashton, 2006). In this measure, each personality scale is measured using 16-items, each measured on a five-point Likert scale (1 = strongly disagree, 5 = strongly agree). Internal consistency reliabilities for both self and observer reports are typically shown to be above 0.70 (Lee and Ashton, 2006).

##### Equity sensitivity

Each participant completed self-reports of the EPQ (Sauley and Bedeian, 2000) and the ESI (Huseman et al., 1985). While the EPQ involves rating a series of statements using a five-point Likert scale (see Study 1 and 2), the ESI takes a somewhat different approach. Specifically, the ESI presents five items which each have two options (“A” vs. “B”). Between the two options in each item, participants are given 10 points, and asked to allocate the 10 points between the two choices by giving the most points to the choice that is most like them and the fewest points to the option that is least like them. They are told they can use as few points as they'd like for any specific option, but must allocate all 10 points in each question. One sample question is: “I would be more concerned about: (A) What I received from the organization, (B) What I contributed to the organization.” The total points allocated to option B are totaled (two of the items are reverse-scored), and higher scores indicate Benevolents, whereas lower scores indicate Entitleds.

### Results

The means, standard deviations, internal consistency reliabilities, and intercorrelations among the study variables can be found in Table 5. Consistent with Study 2 and Hypothesis 5a, self-and peer-reports of Honesty-Humility were positively correlated with scores on both the EPQ (*r* = 0.40, *p* < 0.01; *r* = 0.18, *p* < 0.01, respectively) and ESI (*r* = 0.45, *p* < 0.01; *r* = 0.20, *p* < 0.01, respectively). Consistent with the previous studies, both self and peer-reported Conscientiousness was positively related to scores on the EPQ (*r* = 0.29, *p* < 0.01; *r* = 0.23, *p* < 0.01, respectively). Interestingly, the relation between Conscientiousness and the ESI was not robust, with only peer-reports (*r* = 0.13, *p* < 0.05), not self-reports (*r* = 0.10, ns), relating to the ESI scores. Moreover, the sizes of these relations were much smaller than observed with the EPQ. Both self- and peer-reports of Agreeableness were positively correlated with scores on the ESI (*r* = 0.19, *p* < 0.01; *r* = 0.15, *p* < 0.05, respectively), but neither self- nor peer-reported Agreeableness were significantly related to scores on the EPQ (*r* = 0.10, ns; *r* = 0.11, ns, respectively). Regarding Emotionality, neither self- nor peer-reports were significantly related to scores on the EPQ (*r* = 0.03, ns; *r* = 0.11, ns, respectively) or ESI (*r* = 0.08, ns; *r* = 0.11, ns, respectively) contrary to Hypothesis 3. Regarding Openness, neither self- nor peer-reports correlated with either the ESI (*r* = −0.02, ns; *r* = 0.07, ns, respectively) or EPQ (*r* = 0.03, ns; *r* = 0.05, ns, respectively) scores. Finally, regarding Extraversion, the only significant relation observed was between peer-rated Extraversion and the EPQ (*r* = 0.12, *p* < 0.05). Overall, we found some support for Hypotheses 1, 2, and 5a but not Hypotheses 3 and 4.

**Table 5:** **Variable means, standard deviations, intercorrelations, and Cronbach's alphas for Study 3**.

**Variable**	***M***	***SD***	**1**	**2**	**3**	**4**	**5**	**6**	**7**	**8**	**9**	**10**	**11**	**12**	**13**	**14**
1. EPQ	3.57	0.56	0.87^a^													
2. ESI	25.31	6.31	0.60	0.85^a^												
3. Hs	3.19	0.57	0.40	0.45	0.80^a^											
4. Es	3.46	0.59	0.03	0.08	0.11	0.83^a^										
5. Xs	3.50	0.55	0.11	0.05	−0.09	−0.21	0.85^a^									
6. As	2.92	0.59	0.10	0.19	0.31	−0.19	0.04	0.85^a^								
7. Cs	3.49	0.54	0.29	0.10	0.04	0.07	0.13	0.06	0.82^a^							
8. Os	3.35	0.61	0.03	−0.02	0.06	−0.04	0.16	0.10	−0.01	0.82^a^						
9. Hp	3.19	0.60	0.18	0.20	0.33	0.21	−0.18	0.17	0.03	−0.06	0.85^a^					
10. Ep	3.31	0.63	0.11	0.11	0.17	0.57	−0.19	−0.10	0.14	−0.07	0.05	0.88^a^				
11. Xp	3.53	0.57	0.12	0.07	−0.07	−0.15	0.58	0.02	0.01	0.11	−0.06	−0.16	0.87^a^			
12. Ap	3.10	0.65	0.11	0.15	0.14	−0.07	−0.07	0.47	0.01	0.02	0.34	−0.11	0.08	0.89^a^		
13. Cp	3.49	0.57	0.23	0.13	0.09	0.23	−0.05	−0.05	0.43	−0.07	0.21	0.22	0.12	0.05	0.86^a^	
14. Op	3.08	0.60	0.05	0.07	0.11	0.07	0.08	0.05	0.02	0.54	0.04	0.09	0.17	0.01	0.08	0.84^a^

*M, mean; SD, standard deviation; EPQ, Equity Preference Questionnaire; ESI, Equity Sensitivity Instrument; H, Honesty-Humility; E, Emotionality; X, Extraversion; A, Agreeableness; C, Conscientiousness; O, Openness to Experience; s, self-report; p, peer-report*.

*r > 0.17, p < 0.01; r > 0.12, p < 0.05*.

a *Cronbach's alpha*.

#### Multiple regression analyses

We ran four sets of analyses, namely (a) self-reports of personality predicting self-reports of the EPQ, (b) peer-reports of personality predicting self-reports of the EPQ, (c) self- reports of personality predicting scores on the ESI, and (d) peer-reports of personality predicting self-reports of the ESI. These results can be seen in Table 6.

**Table 6:** **Summary of the multiple regression and relative weight analyses for Study 3**.

**Variable**	**EPQ**					**ESI**				
	**β**	**rRW**	**RW%**	**CI_L_**	**CI_U_**	**β**	**rRW**	**RW%**	**CI_L_**	**CI_U_**
Hs	0.41^**^	0.15	62.21	0.08	0.26	0.43^**^	0.19	80.72	0.12	0.27
Es	−0.02	0.00	0.43	−0.02^a^	0.01^a^	0.06	0.01	2.78	−0.01^a^	0.05^a^
Xs	0.11^*^	0.01	5.27	−0.00^a^	0.06^a^	0.11	0.01	2.84	−0.01^a^	0.05^a^
As	−0.04	0.00	1.84	−0.01^a^	0.02^a^	0.09	0.02	9.96	+0.00	0.07
Cs	0.26^**^	0.07	30.08	0.03	0.15	0.06	0.01	2.90	−0.01^a^	0.05^a^
Os	−0.00	0.00	0.17	−0.02^a^	0.01^a^	−0.06	0.00	0.81	−0.01^a^	0.03^a^
R^2^	0.25^**^					0.23^**^				
Hp	0.12	0.02	23.19	−0.00^a^	0.07^a^	0.15^*^	0.03	38.45	+0.00	0.08
Ep	0.09	0.01	11.11	−0.01^a^	0.06^a^	0.11	0.01	16.55	−0.01^a^	0.06^a^
Xp	0.12	0.02	15.86	−0.00^a^	0.06^a^	0.07	0.01	6.67	−0.01^a^	0.05^a^
Ap	0.07	0.01	8.47	−0.01^a^	0.05^a^	0.10	0.02	21.52	−0.00^a^	0.07^a^
Cp	0.17^*^	0.04	40.30	+0.00	0.10	0.06	0.01	12.70	−0.01^a^	0.05^a^
Op	0.01	0.00	1.07	−0.01^a^	0.03^a^	0.04	0.00	4.11	−0.01^a^	0.04^a^
R^2^	0.09^**^					0.07^**^				

*Raw relative weight; RW%, relative weight percentage; CI_L_ and CI_U_, lower and upper bounds, respectively, of the 95% confidence interval for the significance test; H, Honesty-Humility; E, Emotionality; X, Extraversion; A, Agreeableness; C, Conscientiousness; O, Openness to Experience; s, self-report; p, peer-report*.

** p < 0.01;

* *p < 0.05*.

a *Confidence intervals that contain zero are considered to be non-significant*.

First, in the prediction of the EPQ using self-reported personality measures, Honesty-Humility (β = 0.41, *p* < 0.01), Extraversion (β = 0.11, *p* < 0.05), and Conscientiousness (β = 0.26, *p* < 0.01) were significantly related to scores on the EPQ. Together, the six personality traits predicted 24.8% of the variance in EPQ scores. In the second analysis, the six peer-reported personality traits were regressed on the EPQ measure of equity sensitivity. In this analysis, we found that peer-rated Honesty-Humility (β = 0.12, *p* < 0.05, one tailed) and peer-rated Conscientiousness (β = 0.17, *p* < 0.05) positively relating to EPQ scores. These findings are similar to those obtained from the self-report personality measures. However, peer-reported Extraversion was only marginally related to scores on the EPQ (β = 0.12, *p* < 0.06). In sum, peer-reported personality significantly accounted for EPQ scores, although the proportion of variance was much smaller (9.2%).

In terms of prediction of the ESI, the first analysis (involving self-report personality) indicated that only Honesty-Humility significantly predicted ESI scores (β = 0.43, *p* < 0.01). Together, personality predicted 22.9% of the variance in ESI scores. In the analysis involving peer-reports of personality, again, only Honesty-Humility emerged as a significant predictor of ESI scores (β = 0.15, *p* < 0.05). Peer-reported personality still significantly accounted for ESI scores, although the proportion of variance accounted for was small (7.1%).

##### Relative importance analyses

Results of the relative importance analysis are presented in Table 6. For the first analysis (i.e., self-reports of HEXACO and EPQ), only Honesty-Humility (62.21%) and Conscientiousness (30.08%) accounted for a significant portion of variance in equity sensitivity. For the second analysis (i.e., peer-reports of HEXACO and EPQ), only Conscientiousness (40.30%) accounted for a significant portion of variance in equity sensitivity. For the third analysis (i.e., self-reports of HEXACO and ESI), only Honesty-Humility (80.72%), and Agreeableness (10%) accounted for a significant portion of the total variance in equity sensitivity. For the fourth and final analysis (i.e., peer-reports of HEXACO and ESI), only Honesty-Humility (38.45%) accounted for a significant portion of variance in equity sensitivity. In sum, Honesty-Humility, Conscientiousness, and Agreeableness were found to be integral to the understanding of equity sensitivity across different rating sources and measurement scales, although there was some variability across analyses and sources, as discussed below.

### Discussion

Study 3 was designed to expand on the previous two studies by taking a more comprehensive look at the relations between personality and equity sensitivity. Using the HEXACO framework, we examined (a) whether the relations from the first two studies generalized when using peer-reports of personality and (b) how the relations generalized to another prominent measure of equity sensitivity (the ESI). Although we integrate the findings from this study into the overall picture from the three studies below, we highlight a few important findings.

First, many of the findings from the first two studies generalized to this study and when using peer-reports of personality. In general, the traits of Honesty-Humility, Conscientiousness, and Agreeableness all emerged in some analyses as significant. However, the only personality trait that was robust across self and peer reports of personality as well as across measures of equity sensitivity (EPQ and ESI) was Honesty-Humility. This further illustrates the importance of including Honesty-Humility in understanding the dispositional basis of equity sensitivity. Moreover, the finding that some relations with equity sensitivity did generalize to peer-reports gives further confidence that these relations are not just due to common method factors. Finally, evaluating the *R*^2^-square values for the HEXACO in predicting the EPQ and ESI, it appears that each contains similar overlap with broad personality; however, the ESI appears to be more predominantly related to Honesty-Humility, whereas the EPQ relates to a broader array of traits.

## General discussion

The current investigation consisted of three studies that examined the nomological network of equity sensitivity in regards to the two most prevalent personality models: the Big Five and HEXACO models. We hypothesized that—across both models—Conscientiousness, Agreeableness, and Openness to Experience would be positively related to equity sensitivity, whereas Neuroticism would be negatively related. The hypotheses regarding Conscientiousness and Agreeableness were generally supported across all three studies. For Conscientiousness, the only time this variable was uncorrelated with equity sensitivity was in Study 3 when it was measured with self-ratings and correlated with the ESI. Agreeableness was related to equity sensitivity, with the exception of both self- and peer-ratings of Agreeableness with the EPQ in Study 3. In sum, both Conscientiousness and Agreeableness were positively related to equity sensitivity in at least four of six correlational analyses, indicating fairly robust support for both Hypotheses 1 and 2. In addition, multiple regression and relative weight analyses demonstrated that both contributed uniquely and positively to the prediction of equity sensitivity in many cases. These findings are consistent with previous findings (cf. Konovsky and Organ, 1996; Bing and Burroughs, 2001; Scott and Colquitt, 2007).

We also examined the role of Honesty-Humility—from the HEXACO personality model (Lee and Ashton, 2004)—in understanding equity sensitivity. Although, previous research has not investigated the relation between Honesty-Humility and equity sensitivity, we had strong theoretical and empirical reason to believe that Honesty-Humility would be integral to this construct. We hypothesized that: (a) Honesty-Humility would be positively related to equity sensitivity and (b) it would add to the prediction of equity sensitivity beyond the Big Five personality traits. Support for this hypothesis was found across Studies 2 and 3. In both studies, Honesty-Humility had significant, positive correlations with equity sensitivity and positively predicted unique variance in equity sensitivity beyond the Big Five traits. In addition, relative weight analyses indicated that Honesty-Humility was an important contributor to predicting equity sensitivity, accounting for a significant portion of the variance in the regression models. In sum, the findings regarding Honesty-Humility were consistent and strong, suggesting that individuals who score high on this trait are more likely to be input oriented (i.e., Benevolent) in the workplace. Further, it consistently had large effects indicating that Honesty-Humility was one of the more robust predictors of equity sensitivity in the current investigation.

In regards to the remaining personality traits, the findings for Neuroticism generally suggest a non-relation with equity sensitivity. Although, we hypothesized a modest negative relation between the two constructs on the basis of some limited past research (Raja et al., 2004; Scott and Colquitt, 2007), across all three studies, Neuroticism was only significantly, negatively correlated to equity sensitivity in Study 2. However, Neuroticism did not predict unique variance in equity sensitivity in this study, nor did it account for a significant portion of the variance in the regression model. Coupling these results with the non-significant relations found in Studies 1 and 3, we conclude that Neuroticism does not play a substantial role in understanding equity sensitivity.

The findings for Openness provided limited support of the hypothesis. Although a small, positive correlation was found between Openness and equity sensitivity in Studies 1 and 2, the relation between Openness and equity sensitivity disappeared in the regression models, indicating that Openness did not predict any unique variance in equity sensitivity. Further, Openness was found to be unrelated to equity sensitivity across all analyses in Study 3. We therefore, only found partial support for a positive relation between Openness and equity sensitivity. There are two possible reasons for only finding partial support for Hypothesis 4. First, research by Bolton et al. (2010) found Openness to be positively related to *counterproductive* behavior, a behavior similarly demonstrated by Entitleds (Scott and Colquitt, 2007). Second, Research by Ng et al. (2005) found Openness to have a small, positive relation with salary, suggesting that Openness may have some association with being outcome oriented. These findings indicate that the relation between Openness and equity sensitivity may be more complex than originally theorized.

Due to a lack of theoretical clarity about the role of Extraversion in understanding input- and outcome-oriented behaviors, we proposed a research question regarding its relation with equity sensitivity. The current investigation found that Extraversion was only related to equity sensitivity in one of a possible six correlational analyses. In addition, Extraversion only predicted unique variance in equity sensitivity in Study 3; however, it did not account for a significant portion of the variance in the regression model. Further, previous research has demonstrated that Extraversion can be positively related to equity sensitivity (i.e., more Benevolent; Raja et al., 2004; Colquitt et al., 2006; Scott and Colquitt, 2007). Nevertheless, other research has demonstrated that Extraversion can be related to entitlement (Campbell et al., 2004; Donnellan et al., 2006). Combining these findings with the present results, and a lack of theory suggesting otherwise, it appears that Extraversion does not play a major role in understanding the dispositional nature of equity sensitivity.

In regards to the Big Five and HEXACO models in their entirety, it appears that each model accounts for a significant portion of equity sensitivity. In Study 1 and 2, the Big Five personality traits accounted for 29 and 28% of the variance in equity sensitivity, respectively. Further, the addition of Honesty-Humility in Study 2 accounted for an extra 5% of variance unaccounted for by the Big Five traits. In Study 3, the HEXACO personality model accounted for 25% of the variance when equity sensitivity was measured using the EPQ and 23% of the variance when equity sensitivity was measured using the ESI. However, when personality was measured using peer ratings, the amount of variance accounted for was much smaller (9 and 7%, respectively). In regards to unique contributions, the personality traits of Conscientiousness, Agreeableness, and Honesty-Humility had the most robust relations with equity sensitivity. As such, despite these demonstrated relations, broad personality traits do not entirely encompass equity sensitivity. Thus, more research is needed to investigate other individual characteristics (e.g., attitudes and values) and/or contextual factors (e.g., workplace culture and leadership) that may contribute to our understanding of equity sensitivity and its nomological network.

Study 3 further provided an opportunity to compare the two most prevalent equity sensitivity measures to examine whether the relations found in Study 1 and 2 would replicate across the scales. Our results suggest that these two measures of equity sensitivity are not wholly equivalent. The ESI and EPQ correlated at *r* = 0.60, which, although high enough to indicate they are tapping similar constructs, suggests they possess unique content. As such, it was beneficial that the third study included an additional measure of equity sensitivity. We found that—although Honesty-Humility was significantly and consistently related equity sensitivity across both questionnaires–there were differences in the personality variables associated with the Equity Preference Questionnaire (EPQ) and the Equity Sensitivity Inventory (ESI). Although, if we examine the trends across all studies, a diverse array of traits emerged as related to scores on the EPQ (predominantly Conscientiousness and Honesty-Humility followed by Big Five Agreeableness), the regression and relative weights analyses in Study 3 suggest that the ESI is predominantly associated with Honesty-Humility. However, past studies that have used the ESI (although focused purely on self-report) have found Conscientiousness and Agreeableness to also emerge as correlates of equity sensitivity (Konovsky and Organ, 1996; Scott and Colquitt, 2007). As such, concluding the ESI does not relate to these traits is premature, and requires more research. In sum, although the research indicates that a common dispositional theme across these measures is a relation with Honesty-Humility, additional trait differences between the two measures requires more research.

Although Shore and Strauss (2008) found support for the unidemsionality of the EPQ, Miller (2009) conducted a confirmatory factor analysis (CFA) of the EPQ and found that a bidimensional structure, with separate benevolent (input-oriented) and entitlement (output-oriented) dimensions, had the best model fit across all three studies. These findings, along with theoretical argument (Davison and Bing, 2008), suggest that distinguishing between these two dimensions may help improve our understanding of the relations between equity sensitivity and certain traits. For instance, some traits may be related to a particular aspect of equity sensitivity (e.g., input) but not the other (e.g., output). As such, it was possible that examining the correlations between personality and input-vs. output-oriented factors could elucidate our understanding of the personality-equity sensitivity relation.

We therefore, conducted a CFA on each of the samples in the current investigation and also found that a correlated, bidimensional structure had the best model fit (see Appendix A in Supplementary Material). However, the bidimensional structure did not contribute to the interpretation of the findings of either the correlation or regression analyses; that is, relevant traits were generally positively related to one dimension and negatively related to the other, with little differential relations in terms of size (see Appendix B in Supplementary Material). Therefore, for the sake of parsimony and to be consistent with the majority of previous research on equity sensitivity and how this construct is treated in the literature, we presented only the unidimensional results. Nevertheless, the commensurate findings between the bidimensional and unidimensional analyses may not occur with other variables. Thus, future research needs to consider using a bidimensional approach when investigating the nomological network for equity sensitivity.

### Limitations and future directions

The present research has a few limitations. First, both Study 1 and 2 relied on self-report data, which could lead to inflated correlations due to common method bias (Podsakoff et al., 2003). One of the methods to addressing common method bias resulting from self-reported data is to use cross-source data (e.g., self and peer ratings). Thus, to address this limitation in Study 1 and 2, Study 3 included both self and peer ratings. The inclusion of cross-source data reduced the size of some relations; however, the findings of Study 1 and 2 were still corroborated in many cases, although the overall association between personality and equity sensitivity was smaller than when utilizing self-reports, as expected.

Nonetheless, participants completed the questionnaires at the same time point across all three studies. It has been argued that having cross-time data would also help address possible common method biases in research to avoid possible state related confounds (Podsakoff et al., 2003). With that being said, personality traits included in the current investigation (i.e., the Big Five and HEXACO) are theorized and measured as stable traits and therefore should not be as heavily influenced by time. For instance, Cohen et al. (2013) demonstrated reasonably high test-rest reliabilities for the HEXACO personality traits, and although sparse, the research regarding equity sensitivity provides some support for its stability over time (see Sauley and Bedeian, 2000).

Overall, common method bias is less of a concern when measures have been previously validated and lack domain overlap (Conway and Lance, 2010). Consistent with this argument, the current investigation used only validated measures of each construct. It is worth noting, however, that the results differed slightly between the two equity sensitivity measures used in Study 3. Further, the correlation between the two measures (*r* = 0.60) suggests that, although there is some possible overlap, the two scales are not measuring the exact same construct. This is not a novel finding, as other research has found a similar correlation between the EPQ and ESI (Wheeler, 2007). We therefore, recommend that: (1) future researchers investigating equity sensitivity consider the differences between the measures, and (2) future research is conducted to examine the validity of these measures to address their lack of overlap.

Second, the participants in this study were university students without extensive work experience. Considering that the items for equity sensitivity as specifically about behavior in the workplace (e.g., “I am most satisfied at work when I have to do as little as possible”), concerns might be raised about the generalizability of the current findings to workplace behavior. Although in both Study 2 and 3 the majority of participants reported having some work experience (78 and 95%, respectively), it can be assumed that the majority of the participants' work experience was part-time and may not generalize as well to employees in full-time positions. However, Highhouse and Gillespie (2009) argued that there is a lack of research evidence that suggests behavior reported in a student sample does not generalize to behavior in the workplace. Further, the authors argue that for construct validation and similar research endeavors (e.g., the current investigation), student samples are practical and useful. With that being said, it is still recommended that future research address this issue by utilizing an applied sample to examine whether the findings presented herein generalize to full-time employees.

Third, we conducted a CFA on the EPQ in all three investigations. All three analyses revealed that a correlated, bidimensional structure had the best model fit. However, when we conducted our analyses with the bidimensional approach it did not contribute to the findings, as all the relevant traits produced no differential relations between the dimensions. Although for the sake of parsimony we presented the unidimensional results, we suggest that future researchers consider the bidimensional approach when conducting their analyses. The lack of differential relations found herein notwithstanding, other traits may produce differential relations with each dimension, contributing to our understanding of the equity sensitivity construct.

Finally, one contribution of this study is that it not only informs our understanding of equity sensitivity, it can enhance our understanding of broad personality traits and *why* they relate to certain behaviors. Exploring the explanatory processes underlying the relations between personality and outcomes is of critical importance in our theoretical understanding of how these relations operate. For instance, research shows that the reason why low levels of Honesty-Humility relate to unethical decisions is because these individuals are more likely to utilize moral disengagement (Ogunfowora et al., 2013). In the context of the present study, knowing that individuals high in Honesty-Humility, Conscientiousness, and Agreeableness are more likely to be input-oriented and less sensitive to perceived injustice could help explain why these variables relate to fairness-related criteria. For instance, equity sensitivity may help explain why Conscientiousness and Agreeableness relate to organizational citizenship behaviors (Organ and Ryan, 1995; Graziano and Eisenberg, 1997), while Honesty-Humility relates to deviant behaviors (Lee et al., 2005a). Although these are only examples, the findings of the present study may help us inform the explanatory mechanisms linking personality traits to important behaviors. These mediating mechanisms and processes should be a topic of future study.

### Conflict of interest statement

The authors declare that the research was conducted in the absence of any commercial or financial relationships that could be construed as a potential conflict of interest.

## Footnotes

^1^We conducted CFAs to investigate the dimensionality of the EPQ for this and all subsequent studies and found commensurate results across all three investigations (see Appendix A). These results are further discussed in the General Discussion.
