# Supplementary material for: Examining Equity Sensitivity: An Investigation Using the Big Five and HEXACO Models of Personality
Source: Front Psychol. 2016 Jan 8;6:2000. doi: 10.3389/fpsyg.2015.02000 (PMC4705277; doi:10.3389/fpsyg.2015.02000)
Supplement: Supplementary file 1 [file DataSheet1.docx]

**Appendix A**

| Table 7.  *Summary of the confirmatory factor analyses for Study 1, Study 2 and Study 3.* | | | | | | |
| --- | --- | --- | --- | --- | --- | --- |
|  | Study 1 | | | | | |
| Model | χ^2^ | Δχ^2^ | RMSEA | SRMR | CFI | TLI |
| 1 Factor | 595.28 | - | .097 | .073 | .787 | .754 |
| 2 Factors-Orthogonal | 480.501 | -114.778 | .085 | .194 | .837 | .812 |
| 2 Factors-Correlated | 315.341 | -173.071 | .064 | .050 | .908 | .893 |
|  | Study 2 | | | | | |
| Model | χ^2^ | Δχ^2^ | RMSEA | SRMR | CFI | TLI |
| 1 Factor | 396.092 | - | .084 | .062 | .843 | .819 |
| 2 Factors-Orthogonal | 427.585 | 31.493 | .089 | .203 | .826 | .799 |
| 2 Factors-Correlated | 245.267 | -150.825 | .059 | .047 | .923 | .911 |
|  | Study 3 | | | | | |
| Model | χ^2^ | Δχ^2^ | RMSEA | SRMR | CFI | TLI |
| 1 Factor | 180.678 | - | .053 | .059 | .901 | .886 |
| 2 Factors-Orthogonal | 286.643 | 136.696 | .083 | .186 | .764 | .728 |
| 2 Factors-Correlated | 162.549 | -16.727 | .047 | .058 | .923 | .910 |
| *Note.* Study 1, N = 499; Study 2, N = 411; Study 3, N = 260. | | | | | | |

**Appendix B**

| Table 8.  *Variable means, standard deviations, intercorrelations and Cronbach’s alphas for Study 1 and 2.* | | | | | | | | | | | | |
| --- | --- | --- | --- | --- | --- | --- | --- | --- | --- | --- | --- | --- |
| Variable | M (Study 1) | SD (Study 1) | α (Study 1) | 1. | 2. | 3. | 4. | 5. | 6. | 7. | 8. | 9. |
| 1. EPQ | 3.53 | 0.63 | .89 | - | -.90** | .87** | .42** | .40** | -.05 | .15** | .03 | - |
| 2. ENT | 2.40 | 0.74 | .86 | -.92** | - | -.58** | -.41** | -.39** | .08 | -.15** | -.04 | - |
| 3. BEN | 3.44 | 0.67 | .83 | .90** | -.65** | - | .33** | .33** | -.01 | .15** | .03 | - |
| 4. C | 3.36 | 0.64 | .78 | .45** | -.47** | .34** | - | .28** | -.33** | .12* | .19** | - |
| 5. A | 3.62 | 0.54 | .76 | .32** | -.32** | .26** | .33** | - | -.33** | .24** | .08 | - |
| 6. N | 2.61 | 0.70 | .82 | -.20** | .23** | -.12* | -.32** | -.17** | - | -.05 | -.31** | - |
| 7. O | 3.47 | 0.60 | .75 | .14** | -.09 | .16** | .04 | .23** | -.09 | - | .21** | - |
| 8. E | 3.54 | 0.73 | .88 | .12* | -.12* | .10 | .12* | .05 | -.44** | .28** | - | - |
| 9. H | - | - | - | .39** | -.40** | .30** | .31** | .58** | -.11* | .15** | -.15** | - |
| M(Study 2) |  |  |  | 3.54 | 2.39 | 3.47 | 3.69 | 3.65 | 2.60 | 3.15 | 3.46 | 3.27 |
| SD(Study 2) |  |  |  | 0.58 | 0.68 | 0.60 | 0.50 | 0.44 | 0.53 | 0.49 | 0.49 | 0.61 |
| α (Study 2) |  |  |  | .89 | .86 | .82 | .88 | .84 | .87 | .82 | .87 | .72 |
| *Note*. Study 1 (N = 499) is presented above the diagonal and Study 2 (N = 411) is presented below the diagonal. M, mean; SD, standard deviation; EPQ, Equity Preference Questionnaire; ENT, Entitlement; BEN, Benevolence; C, Conscientiousness; A, Agreeableness; N, Neuroticism; O, Openness to Experience; E, Extraversion; H, Honesty-Humility.  ** *p* < .01. **p* < .05. | | | | | | | | | | | | |

| Table 9.  *Summary of the multiple regression and relative weight analyses for Study 1.* | | | | | | | | | |
| --- | --- | --- | --- | --- | --- | --- | --- | --- | --- |
|  | EPQ | | | ENT | | | BEN | | |
| Variable | β | rRW | RW% | β | rRW | RW% | β | rRW | RW% |
| C | .38** | .15 | 51.54 | -.37** | .14 | 53.94 | .30** | .09 | 45.76 |
| A | .34** | .12 | 41.12 | -.31** | .11 | 40.33 | .30** | .09 | 43.71 |
| N | .19** | .01^a^ | 3.98^a^ | -.15** | .01^a^ | 2.73^a^ | .19** | .01^a^ | 5.56^a^ |
| O | .04 | .01^a^ | 2.95^a^ | -.03 | .01^a^ | 2.64^a^ | .05 | .01^a^ | 4.48^a^ |
| E | .02 | .00^a^ | 0.42^a^ | .00 | .00^a^ | 0.36^a^ | .01 | .00^a^ | 0.50^a^ |
| *R^2^* | .29** |  |  | .26** |  |  | .20** |  |  |
| *Note*. β, standardized regression weight; *R*^2^, squared multiple correlation; rRW, raw relative weight; RW%, relative weight percentage. C, Conscientiousness; A, Agreeableness; N, Neuroticism; O, Openness to Experience; E, Extraversion; EPQ, Equity Preference Questionnaire; ENT, Entitled; BEN, Benevolent.  ***p* < .01. **p* < .05.  ^a^Confidence interval around relative weight contained zero, therefore non-significant. | | | | | | | | | |

| Table 10.  *Summary of the multiple regression and relative weight analyses for Study 2.* | | | | | | | | | | | | | |
| --- | --- | --- | --- | --- | --- | --- | --- | --- | --- | --- | --- | --- | --- |
|  |  | EPQ | | | | ENT | | | | BEN | | | |
| Variable | Block | β_Block 1_ | β_Block 2_ | rRW | RW% | β_Block 1_ | β_Block 2_ | rRW | RW% | β_Block 1_ | β_Block 2_ | rRW | RW% |
| C | 1 | .41** | .38** | .16 | 48.45 | -.39** | -.47** | .15 | 43.51 | .34** | .32** | .11 | 51.10 |
| A |  | .20** | .05 | .05 | 13.61 | -.22** | -.07 | .05 | 13.81 | .14* | .05 | .03 | 12.84 |
| N |  | .04 | .07 | .01^a^ | 1.91^a^ | .01 | -.04 | .01^a^ | 2.84^a^ | .07 | .09 | .00^a^ | 1.43^a^ |
| O |  | .06 | .03 | .01^a^ | 2.10^a^ | .01 | .06 | .00^a^ | 0.48^a^ | .12* | .10 | .02^a^ | 7.99^a^ |
| E |  | .07 | .14* | .02^a^ | 4.65^a^ | -.07 | -.21** | .02^a^ | 4.81^a^ | .06 | .11 | .01^a^ | 4.65^a^ |
| H | 2 |  | .29** | .10 | 29.28 |  | -.37** | .12 | 34.55 |  | .18** | .05 | 22.00 |
| *R^2^* |  | .28** | .33** |  |  | .27** | .34** |  |  | .19** | .21** |  |  |
| ∆*R*^2^ |  |  | .05** |  |  |  | .07** |  |  |  | .02** |  |  |
| *Note*. β, standardized regression weight; *R*^2^, squared multiple correlation; rRW, raw relative weight; RW%, relative weight percentage. C, Conscientiousness; A, Agreeableness; N, Neuroticism; O, Openness to Experience; E, Extraversion; H, Honesty-Humility; EPQ, Equity Preference Questionnaire; ENT, Entitled; BEN, Benevolent.  ***p* < .01. **p* < .05.  ^a^Confidence interval around relative weight contained zero, therefore non-significant. | | | | | | | | | | | | | |

| Table 11.  *Variable means, standard deviations, intercorrelations and Cronbach’s alphas for self and peer ratings in Study 3.* | | | | | | | | | | | | |
| --- | --- | --- | --- | --- | --- | --- | --- | --- | --- | --- | --- | --- |
| Variable | M (Self) | SD (Self) | α (Self) | 1. | 2. | 3. | 4. | 5. | 6. | 7. | 8. | 9. |
| 1. EPQ | 3.57 | 0.56 | .87 | - | -.91** | .89** | .18** | .11 | .12* | .11 | .23** | .05 |
| 2. ENT | 2.35 | 0.65 | .82 | -.91** | - | -.62** | -.18** | -.12 | -.11 | -.08 | -.23** | -.05 |
| 3. BEN | 3.49 | 0.60 | .75 | .89** | -.62** | - | .14* | .08 | .12 | .13* | .18** | .05 |
| 4. H | 3.19 | 0.57 | .80 | .40** | -.42** | .31** | - | .05 | -.06 | .34** | .21** | .04 |
| 5. E | 3.46 | 0.59 | .83 | .03 | -.01 | .05 | .11 | - | -.16* | -.11 | .22** | .09 |
| 6. X | 3.50 | 0.55 | .85 | .11 | -.12 | .09 | -.09 | -.21** | - | .08 | .12* | .17** |
| 7. A | 2.92 | 0.59 | .85 | .10 | -.10 | .09 | .31** | -.19** | .04 | - | .05 | .01 |
| 8. C | 3.49 | 0.54 | .82 | .29** | -.29** | .24** | .04 | .07 | .13* | .06 | - | .08 |
| 9. O | 3.35 | 0.61 | .82 | .03 | -.01 | .06 | .06 | -.04 | .16* | .10 | -.01 | - |
| M(Peer) |  |  |  | - | - | - | 3.19 | 3.31 | 3.53 | 3.10 | 3.49 | 3.08 |
| SD(Peer) |  |  |  | - | - | - | 0.60 | 0.63 | 0.57 | 0.65 | 0.57 | 0.60 |
| α (Peer) |  |  |  | - | - | - | .85 | .88 | .87 | .89 | .86 | .84 |
| *Note*. Peer-ratings are presented above the diagonal and self-ratings are presented below the diagonal. M, mean; SD, standard deviation; EPQ, Equity Preference Questionnaire; ENT, Entitlement; BEN, Benevolence; H, Honesty-Humility; E, Emotionality; X, Extraversion; A, Agreeableness; C, Conscientiousness; O, Openness to Experience.  ** *p* < .01. **p* < .05. | | | | | | | | | | | | |

| Table 12.  *Summary of the multiple regression and relative weight analyses for Study 3.* | | | | | | | | | |
| --- | --- | --- | --- | --- | --- | --- | --- | --- | --- |
|  | EPQ | | | ENT | | | BEN | | |
| Variable | β | rRW | RW% | β | rRW | RW% | β | rRW | RW% |
| Hs | .41** | .15 | 62.21 | -.44** | .17 | 64.36 | .31** | .09 | 57.91 |
| Es | -.15 | .00^a^ | 0.43^a^ | .05 | .00^a^ | 0.47^a^ | .02 | .00^a^ | 1.22^a^ |
| Xs | .11* | .01^a^ | 5.27^a^ | -.12* | .02^a^ | 5.64^a^ | .09 | .01^a^ | 5.04^a^ |
| As | -.04 | .00^a^ | 1.84^a^ | .05 | .01^a^ | 1.77^a^ | -.02 | .00^a^ | 2.12^a^ |
| Cs | .27** | .07 | 30.08 | -.26** | .07 | 27.62 | .21** | .05 | 32.28 |
| Os | -.00 | .00^a^ | 0.17^a^ | .03 | .00^a^ | 0.15^a^ | .03 | .00^a^ | 1.44^a^ |
| *R^2^* | .25** |  |  | .26** |  |  | .15** |  |  |
| Hp | .12 | .02^a^ | 23.19^a^ | -.14* | .02^a^ | 26.08^a^ | .09 | .01^a^ | 18.57^a^ |
| Ep | .09 | .01^a^ | 11.11^a^ | -.09 | .01^a^ | 11.69^a^ | .07 | .01^a^ | 8.77^a^ |
| Xp | .12 | .01^a^ | 15.86^a^ | -.11 | .01^a^ | 12.81^a^ | .11 | .01^a^ | 19.53^a^ |
| Ap | .07 | .01^a^ | 8.47^a^ | -.03 | .00^a^ | 3.87^a^ | .10 | .01^a^ | 17.69^a^ |
| Cp | .17* | .04 | 40.30 | -.17** | .04 | 44.38 | .13* | .02^a^ | 33.75^a^ |
| Op | .01 | .00^a^ | 1.07^a^ | -.01 | .00^a^ | 1.17^a^ | .01 | .00^a^ | 1.70^a^ |
| *R^2^* | .09 |  |  | .09 |  |  | .07 |  |  |
| *Note*. β, standardized regression weight; *R*^2^, squared multiple correlation; rRW, raw relative weight; RW%, relative weight percentage. C, Conscientiousness; A, Agreeableness; N, Neuroticism; O, Openness to Experience; E, Extraversion; EPQ, Equity Preference Questionnaire; ENT, Entitled; BEN, Benevolent.  ***p* < .01. **p* < .05.  ^a^Non-significant confidence interval around relative weight. | | | | | | | | | |
